# Active navigation and meteorological selectivity drive insect migration patterns through the Levant

**DOI:** 10.1098/rspb.2025.0587

**Published:** 2025-06-25

**Authors:** Yuval Werber, Elior Adin, Jason W. Chapman, Don R. Reynolds, Nir Sapir

**Affiliations:** ^1^ Department of Evolutionary and Environmental Biology, University of Haifa, Haifa 3103301, Israel; ^2^ Department of Biotechnology, Tel-Hai College, Upper Galilee 12208, Israel; ^3^ State Key Laboratory of Agricultural and Forestry Biosecurity, College of Plant Protection, Nanjing Agricultural University, Nanjing, 210095, China; ^4^ Centre for Ecology and Conservation, University of Exeter, Penryn, Cornwall TR10 9FE, UK; ^5^ Natural Resources Institute, University of Greenwich Faculty of Engineering and Science, Chatham Maritime, Kent SE10 9LS, UK; ^6^ Rothamsted Research, Harpenden, Hertfordshire AL5 2JQ, UK

**Keywords:** insect migration, flight behaviour, radar monitoring, Levantine Corridor, eastern Mediterranean, Israel, meteorological effects

## Abstract

Insect migration is crucial to many natural processes and human activities, yet large-scale patterns remain poorly understood. On the Mediterranean’s eastern shores lies a 70 km-wide stretch of hospitable habitat between the sea and the Arabian Desert, which we term the Levantine Corridor, extending ~400 km south from Turkey to the edge of the Sahara. We deployed 7 biological radars over 8 years, recording 6.3 million individual large insects (>10 mg) and revealing an important migration route at the nexus of three continents, with over 700 million large insects estimated to cross annually. However, a comparison with European insect migration flows suggests that Levantine insect fluxes are lower than at higher latitudes, challenging the conjecture that the Levantine Corridor acts as a funnel for insect migration as reported for birds. Insects showed strong migratory directionality differing from prevailing wind direction in spring and autumn, with mass migrations separated by periods of weaker movements. Migration intensity strongly depended on the weather, with insects preferentially migrating in seasonally beneficial tailwinds when possible and in warmer temperatures. The study reveals an unexplored insect migration route with implications for food webs, pollination, disease transmission, pest outbreaks and species invasions across West Asia, East Europe and Northeast Africa.

## 1. Introduction

Insect migration displaces huge amounts of biomass, maintains key ecological functions and has far-reaching positive and negative consequences for humanity [1–6]. Despite its importance, the phenomenon is under-researched and considerable further study is required to characterize major migration pathways, particularly outside the Northern Hemisphere temperate zone. Modern radar technology is one of the few methods suitable for documenting flight at the high altitudes where migration mostly takes place [2,7,8]. As radar use becomes widespread and covers increasing portions of the aerial habitat, insect migration flyways and multigenerational population dynamics of migrants can be studied in a global context. Migration flyways (a concept adopted from the ornithological literature [9,10]) are geographical corridors along which multiple species co-migrate between the northern and southern extremes of their annual ranges. Flyways have particular spatial and temporal properties that are important for our understanding of global patterns and processes of insect migration [6,11]. Key elements of the flyaway are regions where large numbers of migrants are concentrated/funnelled through relatively restricted areas of suitable habitat surrounded by inhospitable areas [4,7,12]. Insect migration through such regions of a flyway may have significant impacts on various ecosystem processes over immense geographic expanses and exert important influences on agricultural yield, disease spread and conservation [2,3,6,13].

Insect migrants, being poikilothermic, small and slow flying, are greatly affected by weather conditions, particularly temperature and winds. Some larger species are known to time their migrations to coincide with favourably directed tailwinds and they adjust their flight headings to achieve transport in seasonally beneficial directions [1,2,14,15]. Coupling meteorological measurements and reanalysis data with precisely documented insect activity offers a unique opportunity to characterize key properties of this important insect migration system, as well as to study decision rules and meteorological preferences among insect migrants.

**Figure 1.**
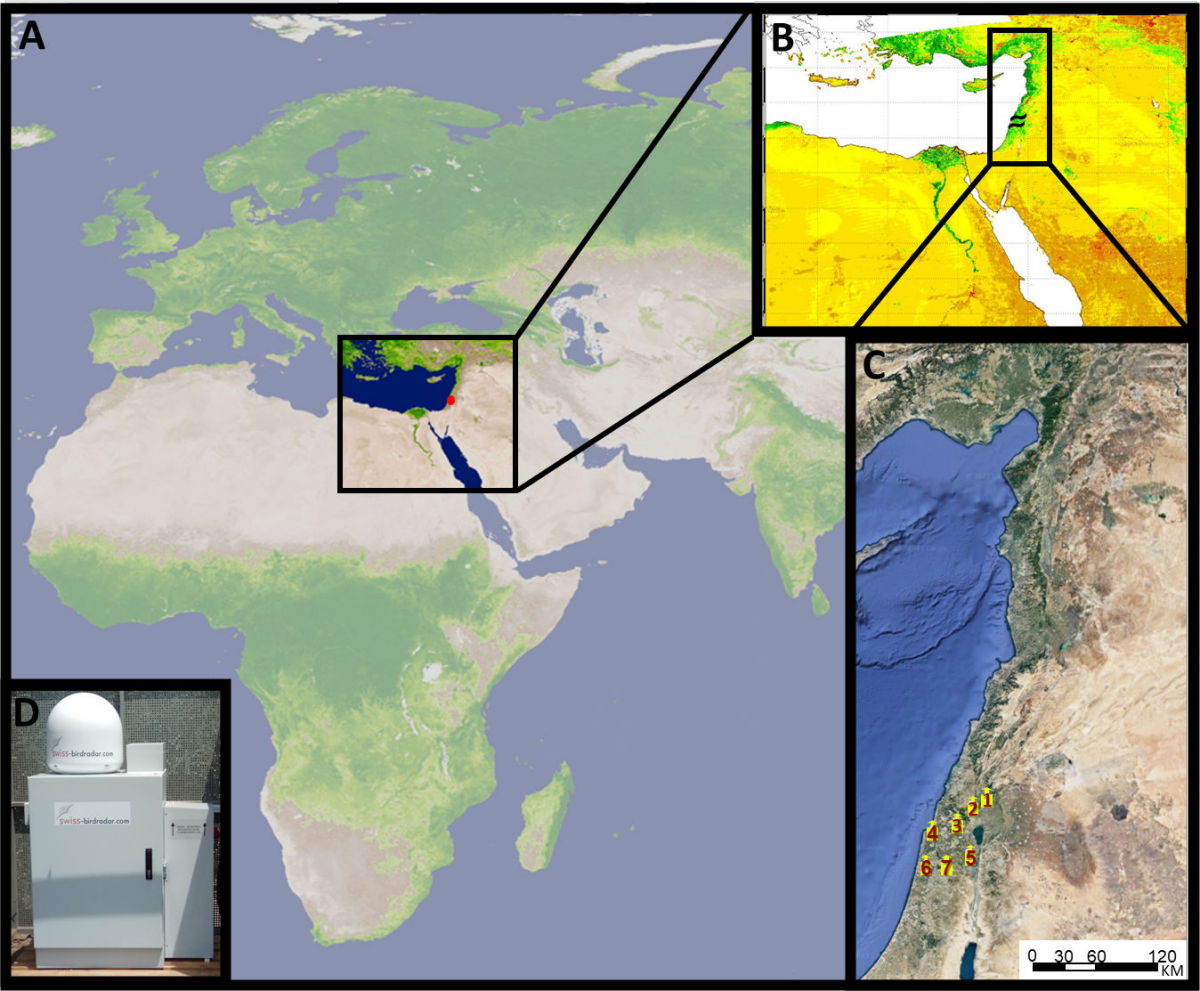
A: Continental perspective of the flyway, constituting a junction between African, Arabian and Mediterranean winter breeding areas and East European/West Asian summer breeding grounds, wedged between sea and desert. The red dot marks our campaign area on the productive Levantine Corridor. B. Regional NDVI(TOA) (normalized vegetation index (top-of-atmosphere)) measurements during the autumn migration period (measured 22 November 2011), emphasizing the difference between the Levantine Corridor and its surroundings. Black wavy lines depict radar deployment locations. C. Satellite image of the Levantine Corridor. Yellow markers indicate radar deployment locations; numbers indicate the north–south order of the sites (1: N.G, 2: Hula, 3: E.U.G, 4: W.G, 5: E.L.G, 6: S.C, 7: J.V) as given in electronic supplementary material, table S1. D. The BirdScan MR1 vertical-looking radar.

The Levant, on the Mediterranean’s eastern shores, constitutes a land bridge between Northeast Africa, West Asia and East Europe (figure 1). Owing to its geographical location, the region plays a key role in global-scale biological movement processes of Afro-Eurasian biogeography [9,16,17]. For example, the Levantine skies—an important aero-ecological bottleneck situated midway along the eastern branch of the African–Eurasian avian flyway system—are a scene of spectacular concentrations of long-range biological movement [18]. Avian migration is well documented throughout the region, but insect migration has mainly been subject to sporadic and local investigations [19–24]. We used a strategically placed network of vertical-beam biological radars (figure 1) to study the high-altitude migration of individual large insects (mass >10 mg) along the ‘Levantine Corridor’, a critical pathway along what we term the ‘East Mediterranean Insect Flyway’, where migrants experience suitable terrestrial habitat and weather conditions, while surrounded by potentially lethal regions.

The Levantine Corridor is situated between the Mediterranean Sea to the west, Arabian Desert to the east and the Saharo-Arabian desert belt to the south, sandwiched between thousands of square kilometres of hostile environment (figure 1). It forms an approximately 400 km-long and 70 km-wide favourable passageway, linking seasonally favourable breeding habitats to the north and south. For many insects migrating through the region, arid desert environments are inhospitable [25] and crossing the eastern Mediterranean Sea is hazardous [4,12]. Thus, the likelihood of completing migratory journeys between Africa, Arabia and Eurasia is higher for insects that migrate within the mild, productive Mediterranean ecosystem along the narrow coastal zone (figure 1). For many insects coming northwards from Northeast Africa and the Arabian Peninsula, the corridor might be the first hospitable terrestrial environment and possible breeding site [17] that they encounter after flying many hundreds of kilometres through extreme desert environments. For insects coming southwards from East Europe/West Asia, the corridor might funnel migration away from the sea or desert and act as an optimal steppingstone ahead of crossing the Sahara or Arabian deserts.

There is an extensive but anecdotal literature comprising visual observations and light-trapping results of (mainly) Lepidopteran movements in the region [19–24,26,27]. However, these studies lacked large-scale context, were typically of short duration, unsystematic and unable to assess movement at the high altitudes where much insect migration takes place [3]. Here, we use ~6.3 million individual insect observations collected by 7 radar deployments across 8 years to quantify intensity, timing and directions of insect migration through the Levantine Corridor and the meteorological drivers of these patterns. Given the geographic context and favourable climate in this region, we hypothesize that repeated, seasonally directed, large-scale movements will occur along this corridor. Owing to the potential concentrating effect of the narrow region of suitability, we predict that, as is the case for birds [10,28], the density of migrants will exceed migration rates measured further north (in Europe) and that autumn returns will tend to be larger than the advance northwards during spring. Large-scale insect migration through the region is likely to be ecologically, economically and epidemiologically important [13,29–32]. Our study thus lays the foundation for in-depth research into this relatively unquantified insect movement, allowing insightful comparisons with important flyways in other parts of the world, e.g. West Europe [25,33–35], Central Europe [36], East Asia [6,37] and North America [11,38].

## 2. Methods

### (a) Hardware and software

Vertical-beam biological radars detect individual animals aloft and gather information regarding their size, shape and movement. They provide a unique view of the aerial environment, and are extensively used for monitoring, research and conservation of flying organisms [2,5,39–41]. Individual measurements of flight direction, shape, speed and wingbeat frequency (WBF) make it possible to analyse large-scale migratory movements with fine resolution and identify migrants as insects, birds or bats [42,43].

The BirdScan MR1 (https://swiss-birdradar.com/systems/radar-birdscan-mr1/) is an X-band vertical-looking pulse radar (9.4 GHz, 25 kW) that is extensively used to monitor flying vertebrates [5,42,44], and increasingly migrating insects [5,45]. It differs from purpose-built entomological radars [7] mainly by having a much wider beam aperture, resulting in shorter detection ranges (nominally 30−500 m above the ground) and longer target acquisition durations. The MR1 wavelength of 3.2 cm limits its insect detection ability to the categories of ‘medium’ and ‘large’ insects as defined by Hu *et al.* [2]. These insects, weighing 10 mg or more, comprise the vast majority of directional insect migrants, even though they typically account for only 0.5−2.5% of all insects moving in the airspace [2,6]. Smaller insects (<10 mg) such as aphids did not show any directional tendency during the same migration period in the airspace examined by Hu *et al.* [2]. Given that these small insects will be carried downwind, and that the prevailing winds in the area blow predominantly eastwards year-round, they are unlikely to form a significant part of the bidirectional, north–south seasonal flow along the Levantine Corridor.

The radar’s software features a classifier that uses echo characteristics to classify targets as insects, several classes of vertebrates and non-biological reflectors [43,46]. Insect classification is based on WBF (automatically identified by the radar’s software), assumed size (based on detection altitude and echo intensity calculated as radar cross section, RCS) and target polarity-based object shape (as the antenna rotates, long-bodied insects like Lepidopterans produce characteristic, sinusoidal modulations because the linearly polarized emission encounters different aspects of the elongated body; these are superimposed on WBF and noise modulations) [46]. More refined body shape parametrizations were unobtainable from our older datasets and were not used in the analysis. Where shape is mentioned, it refers to target polarity-based assessments.

### (b) Data collection

Our dataset was compiled using seven BirdScan MR1 radar deployments spanning the width of the Levantine Corridor section of northern Israel (figure 1). The Hula radar station has been in continuous operation since August 2018; the other six radars were deployed during two bird migration seasons each (during 2015−2018) to assess the bird collision risk at prospective wind farm sites. A detailed description of the different deployments is given in electronic supplementary material, table S1.

The analysis was conducted on radar targets classified as insects (*n* = 6,352,619) based on robust insect classification founded on low overlap in size, body polarity and WBF of vertebrates and insects [7,42,47–52]. Every detection receives a probability factor ranging from 0 to 100 for each class. In order to remove bird detections that were classified as insects from our dataset, we summed these probability factors and excluded echoes with a cumulative bird probability factor larger than 50% (~1.6% of the dataset). We further excluded insect detections with WBF < 10 (~2.5%), given the rarity of insects in this range and the high probability of confusion with vertebrate targets. We only used data from targets beyond 50 m above ground level to avoid ground clutter (and possible non-migratory ‘foraging’ movements of insects), and below 500 m, which is the upper limit of insect-sized target detection achievable with MR1s [45].

Data were collected in short pulse operation mode (70 ns), giving a nominal resolution in range (i.e. height) of ~10 m, operating 40 min out of every hour in all deployments. The antenna was set to rotate 50% of this collection time, which allowed target displacement direction and shape-related polarization information to be collected.

### (c) Meteorological factors

Insect detections were coupled with their closest meteorological reanalysis estimations (wind parameters, temperature, humidity, cloud cover, vorticity and vertical air movement) from European Centre for Medium-Range Weather Forecast (ECMWF)'s ERA5 model (hourly temporal resolution, 0.25° × 0.25° spatial resolution (roughly the area of the Hula Valley) and ~100 m vertical resolution) [53]. These were averaged to create daily/nightly weather metrics for each location to serve as predictors in a boosted regression tree (BRT) framework to determine their relative effects on migration traffic rate (MTR). Sunrise and sunset times, used to define day and night, were set using the ‘sunrise’ and ‘sunset’ functions of the ‘bioRad’ R package [54].

The relationships between MTR and meteorological parameters were analysed with BRT models using the gbm.step function in the R ‘dismo’ package [55]. Each day/night–spring/autumn combination was modelled separately, amounting to four models overall. Log(MTR + 1) was set as the response variable and weather parameters, date and site as predictors. Pairwise correlations among predictors were examined and were found to be generally low. The model used a Gaussian error distribution, and hyperparameters were tuned using grid search to 10-fold cross validation, tree complexity of 12, learning rate of 0.01, bag fraction of 0.75 and a minimum of 25 trees per step.

### (d) Data processing

Processing, analysis and graphics were performed using R Statistical Software (v. 4.1.2 [56]) and R-based statistical packages cited in the text. Where *t*-tests are used, normality was confirmed using the Shapiro–Wilk test, and in cases where data were not normally distributed the Mann–Whitney *U*-test was implemented. Two-tailed tests with *α* = 0.05 were used throughout the analysis.

## 3. Results

### (a) Insect migration seasonality

**Figure 2.**
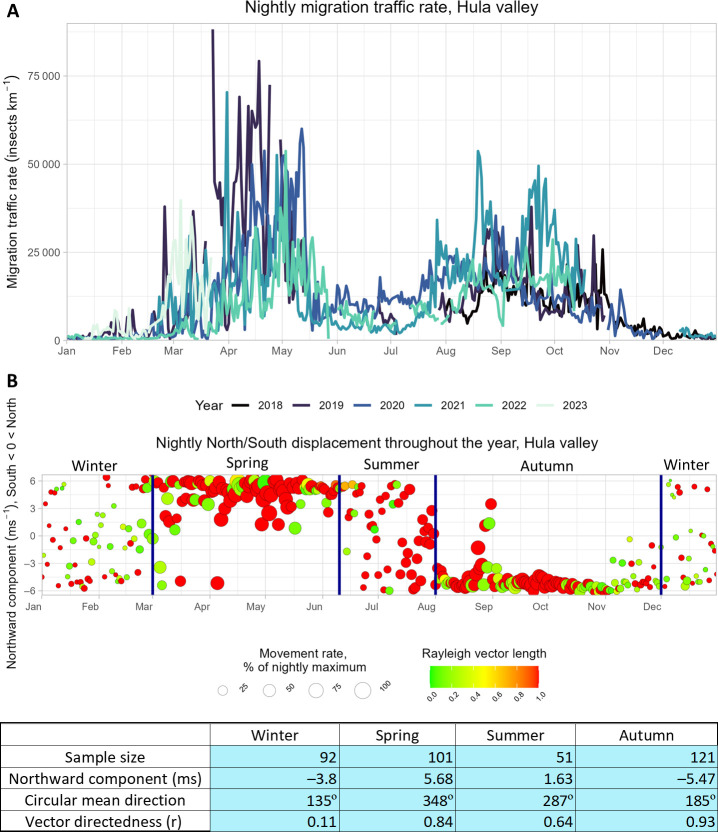
Biological seasonality of nocturnal insect migration movement in the Hula Valley. A. Nocturnal multi-year phenology of the Hula Valley. Nightly (sunset to sunrise) MTR per kilometre above the Hula was calculated for each night across the Hula campaign, showing two seasonal peaks occurring repeatedly across years. B. Each point represents the mean MTR and north–south directionality per night of a specific calendar day across the Hula radar monitoring period (2018−2023). Point size accords with nightly MTR, vertical location refers to average northward component of insect displacement (upwards = north, as in [42]). Point colours indicate nightly Rayleigh vector length (*r*) , reds indicating tightly clustered distributions of individual directions on that night. Spring and autumn migration seasons have strong migration intensity, clear seasonal directionality and overall low directional variability. In the table, the directional statistics are the result of applying Rayleigh tests on the average nightly directionalities for each season. A parallel plot for daytime movements is given in electronic supplementary material, figure S1.

Directionality and volume of insect movement through the Levantine Corridor showed a clear annual rhythm creating four discernible seasons, as illustrated by the nocturnal patterns in the Hula Valley (figure 2). Equivalent daily patterns (electronic supplementary material, figure S1) were generally similar though less clearly defined, likely owing to larger numbers of local movements and stronger effect of W–E winds. Spring and autumn migration periods (defined here as 1 March–10 June and 1 August–30 November, respectively) are characterized by high migration intensity (larger symbols) and significantly directed movements (red colours) matching seasonally expected migratory directions (i.e. northwards in spring, southwards in autumn) (figure 2b). Migration intensity was more instrumental in determining spring migration boundaries, while directional strength (evident in the tight clustering of circles at the bottom of the grid) clearly demarcated autumn migration. Winter (1 December–29 February) and summer (11 June–31 July) tend to have low daily/nightly traffic rates, weak nightly circular directionality (yellows, greens) in winter and uniform scatter across the N–S directional axis (figure 2) in both seasons.

### (b) Insect identity, flight altitude and ground speed

**Figure 3.**
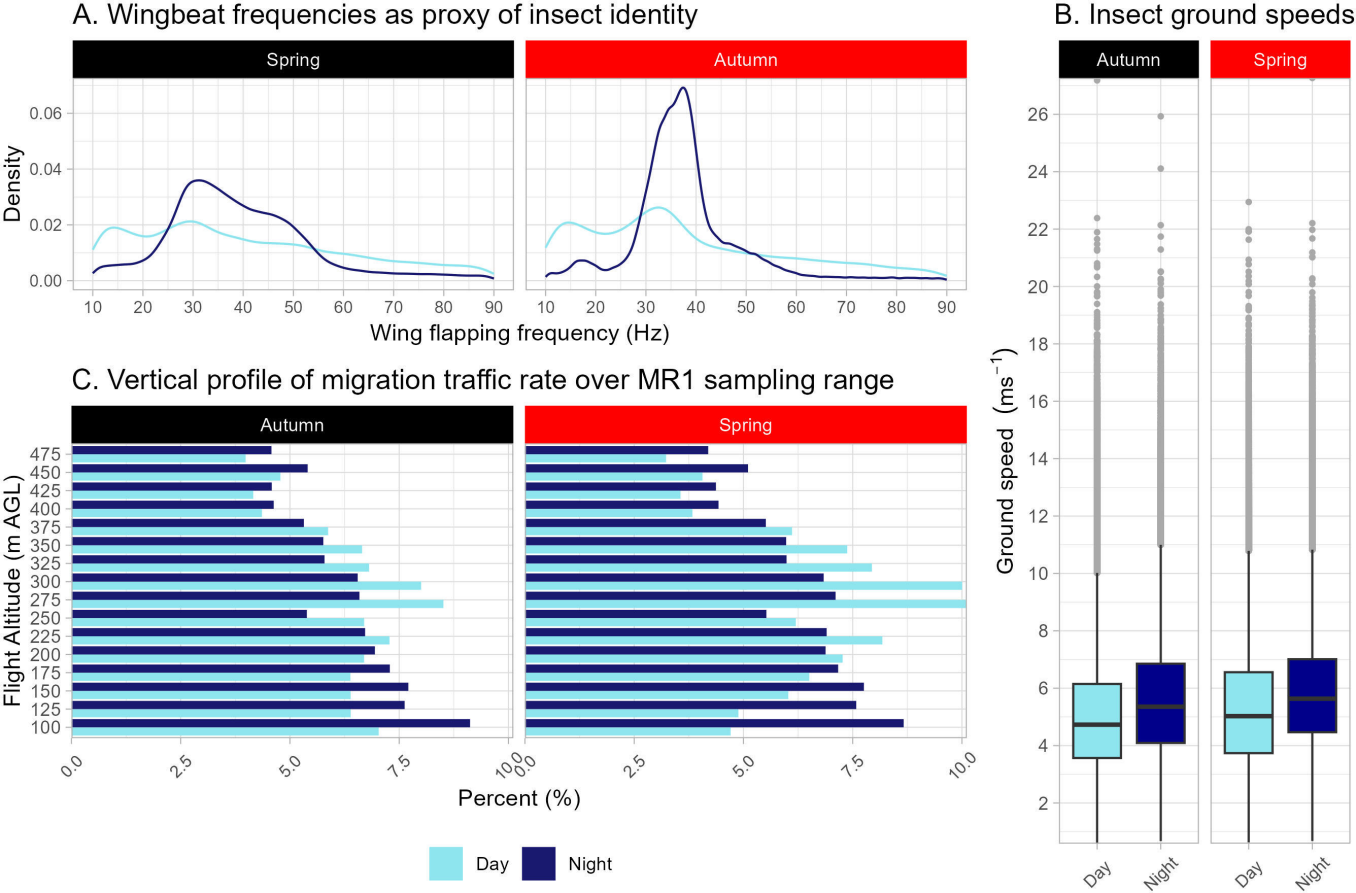
Movement characteristics of insect migration through the Levantine Corridor. A. WBF distributions of detected insect migrants during migration seasons. There are two daytime peaks, ~15 and ~30 Hz, both shallow, in both seasons, corresponding to butterfly and dragonfly WBFs, and a nocturnal peak at 25−50 Hz corresponds to large- to medium-sized moth WBFs. B. The ground speeds of flying insects during migration. Nocturnal flight is significantly faster compared with daytime flight, and spring migration speed is significantly faster than autumn migration speed. Box middle lines represent median values, edges represent quartiles, whiskers extend to the lowest or highest values up to 1.5× the interquartile range and outliers are in grey. C. Vertical profile of insect migration between 100 and 500 m above ground: nocturnal migration is stronger at lower altitudes while daytime passage is higher up.

Within taxonomic groups, WBF is strongly correlated to animal size [49–51,57,58] and, in radar biology, is often used to indicate taxonomic affiliation [5,40,43]. Prominent daytime WBF peaks at 20−40 Hz are produced by large insects, vary across sites and seasons and likely include: large migratory butterflies [59], of which the most likely species are painted lady (*Vanessa cardui),* red admiral (*Vanessa atalanta*), large white (*Pieris brassicae*) and plain tiger (*Danaus chrysippus*) [4,15,33–35,60,61]; dragonflies [59] of migratory species common to the area (e.g. vagrant emperor *Anax ephippiger*, lesser emperor *Anax parthenope*, southern migrant hawker *Aeshna affinis* and red-veined darter *Sympetrum fonscolombii* [4,62,63]); and perhaps grasshoppers (figure 3A). Above this WBF range, for example, at 60−80 Hz, targets are produced by medium-sized insects [59] such as hoverflies, ladybirds and carabid beetles [4,39,64]. The strong peak in nocturnal WBFs (25–50 Hz) likely corresponds to a range of migratory moths [59] in the families Noctuidae, Sphingidae and Crambidae. A concentrated period of light-trapping in the vicinity of the Hula radar indicates that the most important components of the migrant moth fauna in this study were noctuids in the genera *Spodoptera*, *Helicoverpa*, *Heliothis*, *Mythimna* and *Agrotis* (electronic supplementary material, table S2), all of which are known to be important migratory crop pests [1,6,31,59] (figure 3A).

The overall mean insect ground speed was 5.6 ± 0.9 m s^−1^ (figure 3B). Insects travelled slightly faster during the night across all sites in spring (median_day_ = 5.5 m s^−1^, median_night_ = 5.9 m s^−1^, *n*_day_ = 600, *n*_night_ = 608, *t* = 7.6, *p* < 0.0001) and autumn (median_day_ = 5.1 m s^−1^, median_night_ = 5.6 m s^−1^ , *n*_day_ = 655, *n*_night_ = 646, *t* = 12.9, *p* < 0.0001), as similarly reported elsewhere [2]. Ground speeds were faster in spring compared with autumn during day (*t* = 9.3, *p* < 0.0001) and night (*t* = 8.1, *p* < 0.0001).

Activity was spread quite evenly across the detection altitude (50–500 m), although consistent trends did emerge. During the day, activity peaks occurred at 280−320 m above ground, but at night, peaks occurred near the lowest detectable heights (below 150 m) above ground and numbers gradually decreased with altitude (figure 3C).

### (c) Migration traffic rates

Mean daytime MTR averaged across all locations (mean: 24 393 insects km^−1^ day^−1^; 10−90 percentiles: 2167–48 913) was ~14% lower compared with the nightly rate in spring (28 393 insects km^−1 ^night^−1^; 4923−62 572; electronic supplementary material, figure S2) and ~5% lower in autumn (18 969 insects km^−1^ day^−1^; 4833–37 477; 19 923 insects km^−1^ night^−1^; 6159–36 353; electronic supplementary material, figure S2).

Migration was significantly more intense during spring compared with autumn when averaged across all locations (Wilcoxon tests; day: *p* < 0.0001; night: *p* < 0.0001) and at most locations during daytime and night-time (figure 2, electronic supplementary material, figure S2). Day-to-day variation in MTRs is high (note the large range of 10−90 percentiles above) and isolated mass migration events (sudden peaks in MTR preceded and followed by substantially lower values) were common across sites (electronic supplementary material, figure S2). Extrapolating from these figures across the width of the Levantine Corridor (~70 km), the mean seasonal MTRs of radar-detectable insects (i.e. those with body mass >10 mg, assumed to have an average mass of 50 mg according to [2]) were 376.9 million/70 km corridor/season in spring (corresponding to ~19 tonnes of biomass), moving largely northwards, and 332.1 million/70 km/season in autumn (88% of the spring passage, corresponding to ~16 tonnes of biomass), moving southward (electronic supplementary material, table S3). Biomass estimations are based on assumptions of body mass distributions taken from UK-based studies [2] and thus require local validation.

### (d) The directionality of insect movements

**Figure 4.**
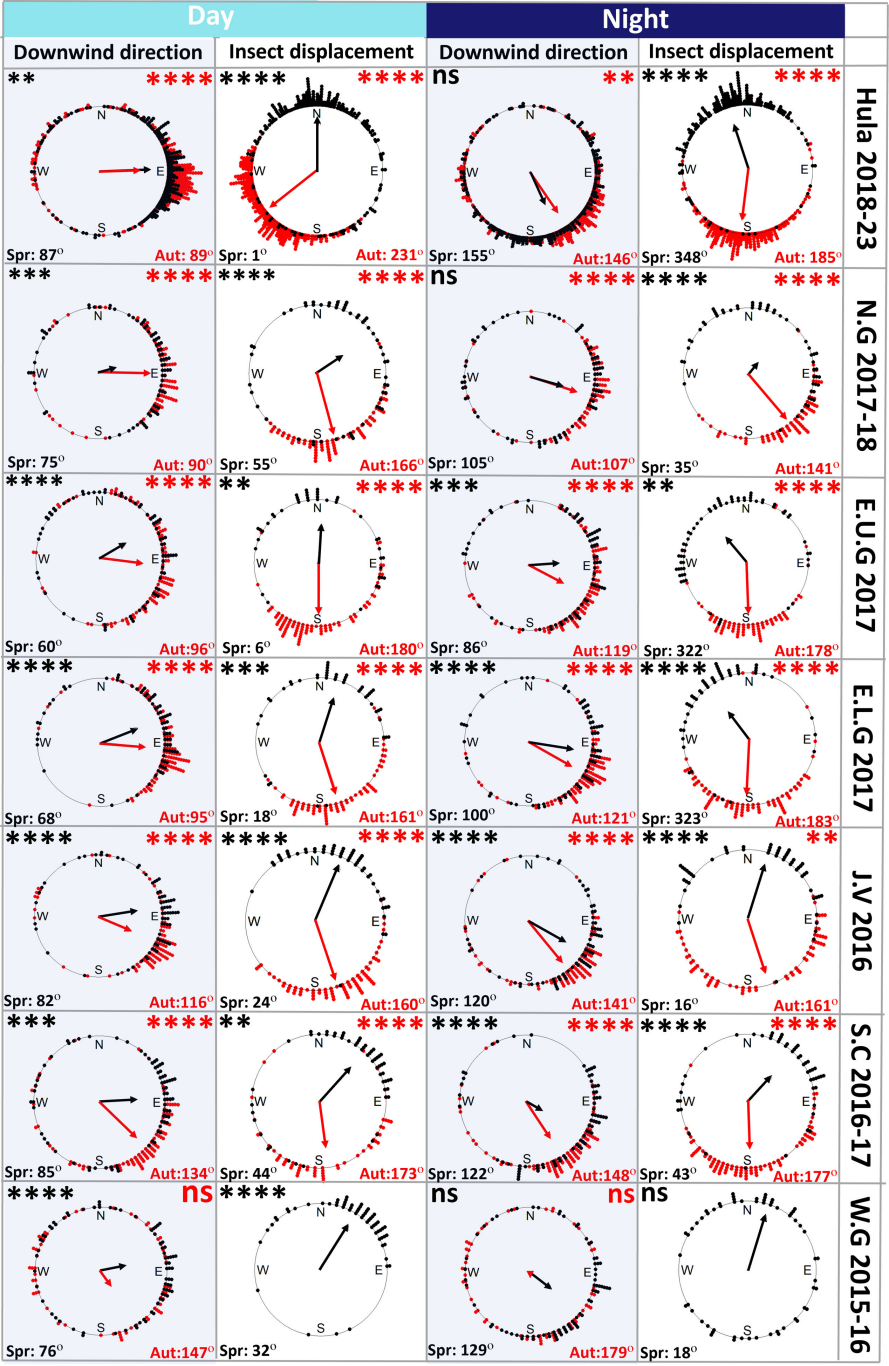
Insect displacement and downwind directions across sites, seasons and years. Insect directionality (white background) and downwind direction (grey background) circular plots for each site, day and night. Black dots represent spring days/nights and red dots represent autumn days/nights. Each dot is placed on the periphery of the circle according to the average direction of insect movement or downwind flow during each day/night. Only the days/nights when the directionality of insect movement was statistically significant are included. Arrow directions indicate the average insect displacement/wind direction (indicated below the plot) and arrow lengths represent the Rayleigh test vector lengths. Asterisks represent the significance levels of average seasonal directions (*p < 0.05, **p < 0.01, ***p < 0.001, ****p < 0.0001) from Rayleigh tests applied for each season/location subset of insect displacement and downwind direction data. Plots of wind direction at flight altitude were based on ECMWF ERA5 model output. Full directional statistics are given in electronic supplementary material, table S4, and radar site codes are listed in electronic supplementary material, table S1.

Mean seasonal movement directions in the Levantine Corridor accorded with expected migratory directions for both seasons (average displacement direction through the corridor across all sites in Spring—day: 2° ± 95°, night: 338° ± 96°; autumn—day: 205° ± 77°, night: 192° ± 92°) and were mostly highly significant (figure 4, white columns titled ‘Insect displacement’). The prevailing wind direction is from the west/northwest (i.e. blowing towards the east/southeast, Spring—day: 63° ± 89°, night: 152° ± 89°; Autumn—day: 98° ± 71°, night: 142° ± 52°) in both seasons (figure 4, grey columns titled ‘Downwind direction’). Insect displacement direction was significantly different from downwind direction at flight altitude taken from ECMWF’s ERA5 model [53] during both migration seasons during day (Watson’s *U*^2^ test: Spring: *U*^2^ = 6.39, *p* < 0.001; Autumn: *U*^2^ = 20.83, *p* < 0.001) and night (Spring: *U*^2^ = 9.02, *p* < 0.001; Autumn: *U*^2^ = 5.99, *p* < 0.001). Despite discordance between prevailing downwind direction and the seasonally beneficial migration direction across sites, seasons and daily periods (electronic supplementary material, table S4), insects managed to migrate northwards in spring and southwards in autumn (figure 4, white columns), confirming that larger insects intentionally navigate in seasonally beneficial directions through the Levantine Corridor. Directionality was most pronounced in the Hula Valley—the lowest-elevation site—and least pronounced at Eastern Upper Galilee and Northern Golan, both comprising the highest-elevation sites, as seen in the arrow lengths which depict the *r*-values (indicating to the degree of clustering of the individual directions around the mean; figure 4). Interestingly, the distance between the Hula radar and these sites is less than 25 km.

**Figure 5.**
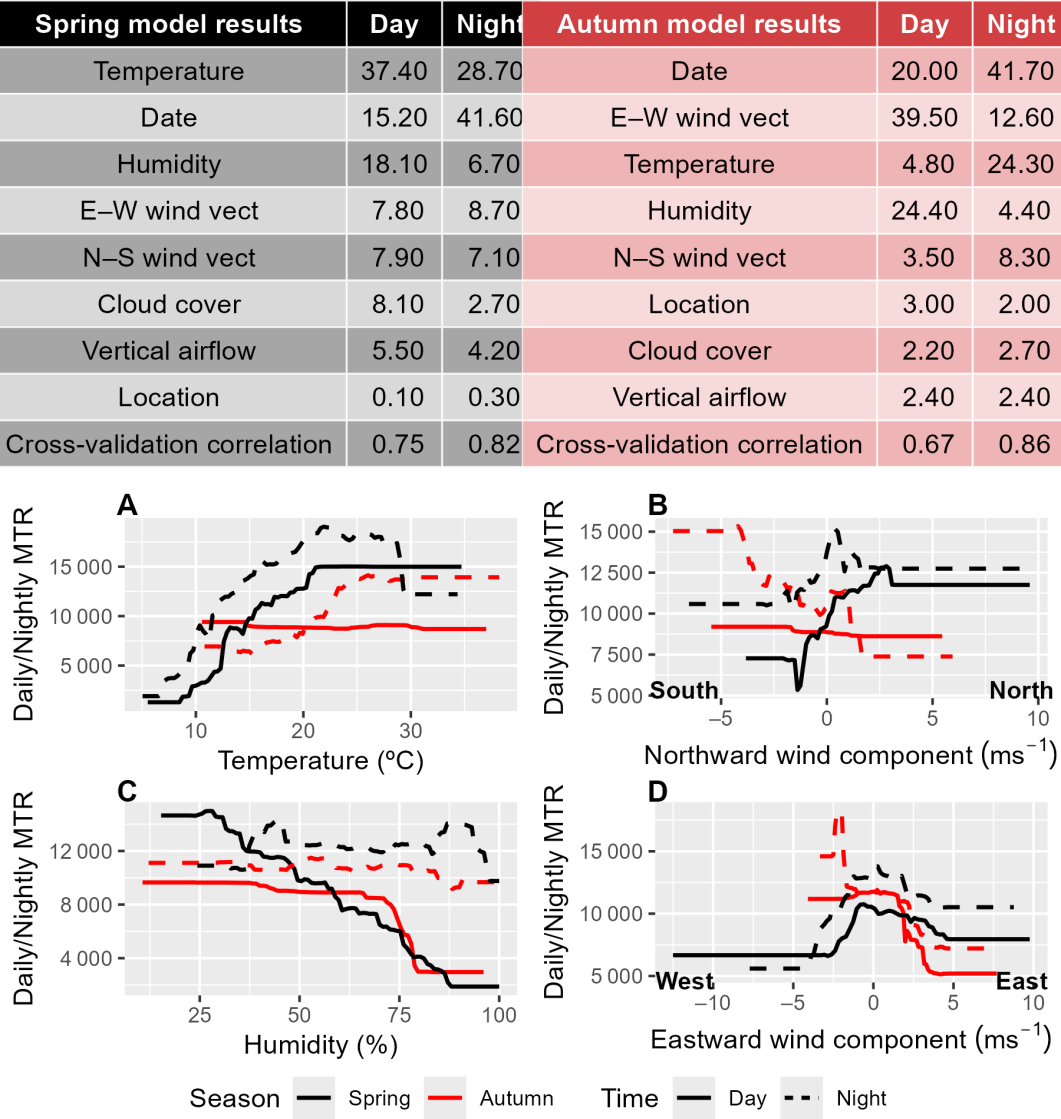
Meteorological factors affecting insect migration traffic rate. Boosted regression tree (BRT) outputs for seasons and day/night. The table specifies the relative importance (>1) of major weather parameters in the model. Higher numbers indicate that a parameter explains a greater portion of the variance in MTR. Since each column summarizes a different model (Season–daynight combination); the relative importance is not directly comparable between models but the order of importance is. Cross-validation correlation is a proxy of model efficiency in capturing MTR variance. Plots describe the predicted relationships between major meteorological parameters and MTR. Specified direction labels at the bottom corners of sub-plots (B) and (D) indicate the direction towards which the wind component is blowing: (B) ‘North’—wind blowing into the corridor from Northeast Africa and through the corridor towards East Europe/West Asia; ‘South’—wind blowing from East Europe/West Asia, through the corridor, towards Northeast Africa; (D) ‘East’—wind blowing from the Mediterranean Sea through the corridor towards the Syrian Desert; ‘West’—wind blowing from the Syrian Desert through the corridor towards the sea.

### (e) Weather determinants of migration intensity

Boosted regression tree models were used to analyse relationships between insect MTRs and key meteorological factors (figure 5). BRT results reveal that migrating insects are highly responsive to meteorological conditions, as evident from the high cross-validation correlation values (figure 5).

In spring, temperature is the most important covariate: insects show strong positive relationships between MTR and temperature, especially at night when temperatures will be more limiting for flight (figure 5A). The northward wind component is also relatively important, with more insects flying in southerly winds (figure 5B). Humidity is an important predictor mainly during the day, when there is a strong negative relationship (figure 5C).

In autumn, the eastward wind component is the most important meteorological factor. Our model predicts virtually no migration along the corridor in autumn when strong winds are blowing from west to east (figure 5D), i.e. from the Mediterranean Sea. Temperature and humidity are next in importance with hot, humid conditions keeping migrants on the ground during the day, with a preference to migrate on warmer nights. Finally, autumn migration is predicted to decrease as the northward component of the wind increases (figure 5B), meaning insects avoid headwinds and utilize tailwinds as they migrate southwards.

### (f) Comparison with migration intensity at higher latitudes

**Figure 6.**
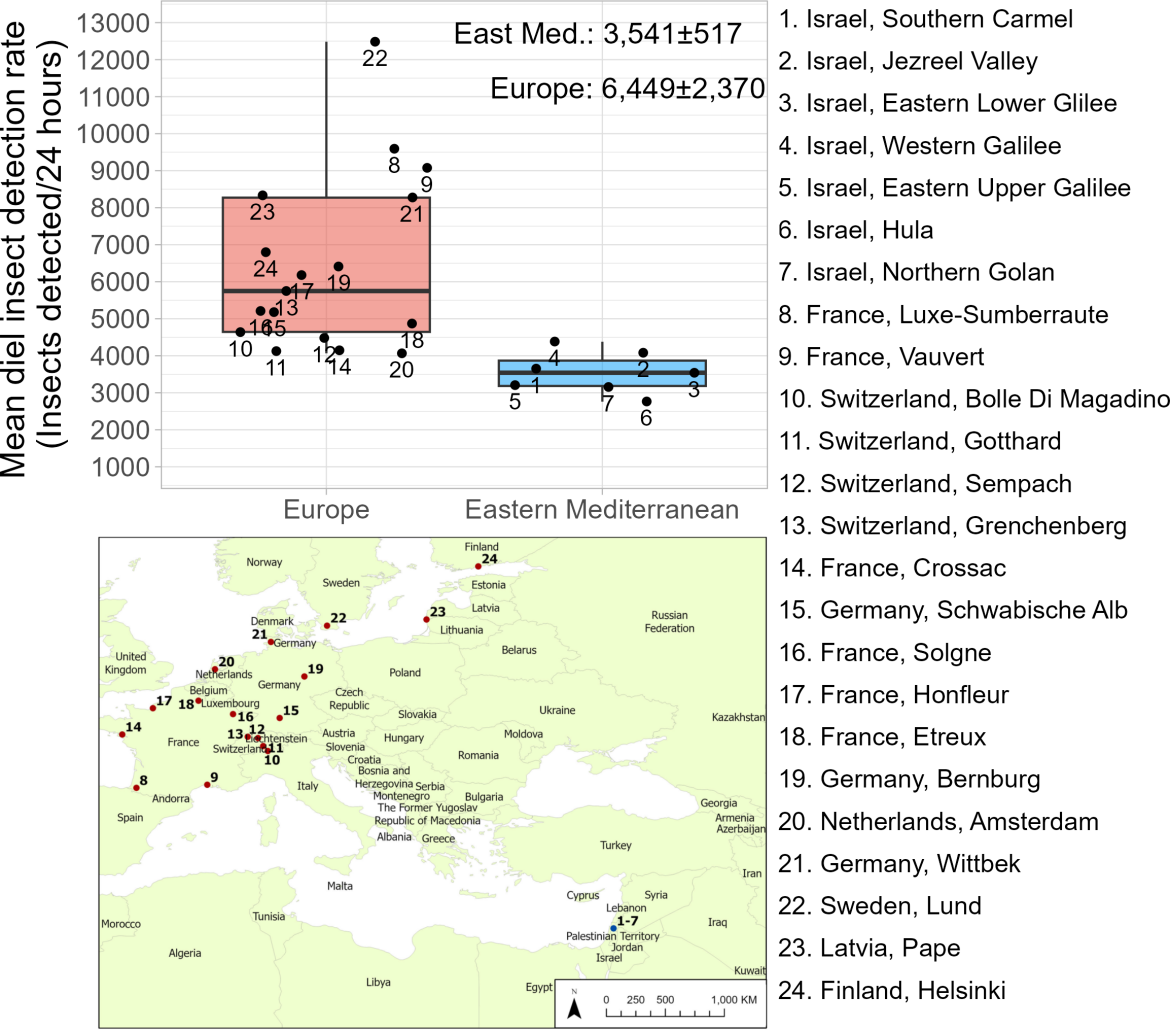
Comparison of mean diel insect detection rates between Europe and the East Mediterranean. Box middle lines represent median values, edges represent upper and lower quartiles and whiskers extend to the lowest or highest value up to 1.5× interquartile range. European detection rates are considerably higher.

To assess the scale of migration through the Levantine Corridor and test our hypothesis that this region acts as a funnel, we calculated mean diel insect detection counts (counts were used to maintain comparability with [45]; see electronic supplementary methods for details) across all locations in Israel from March to October 2015−2023. We then extracted comparative insect counts from 17 European locations (figure 6, 8–24) where BirdScan MR1 radars were also used to monitor aerial abundance of insects during March–October 2021 [45]. The European radars had slightly different filtering procedures in place compared with the Israeli radars, but they were otherwise operationally identical and we do not believe this small difference will strongly affect the numbers of insects detected by the systems. Surprisingly, mean daily insect counts were much lower at all Israeli sites than at all European locations (figure 6) and the overall average in the Levantine Corridor was only approximately half of the overall value across Europe (mean Israeli detection rate: 3541 ± 517 insects per day; mean European detection rate: 6449 ± 2370). The data thus strongly indicate that while insect migration through the Levant is substantial, it is considerably smaller than in the sites further north (contrary to our *a priori* expectations) that are potential destinations of spring migration and potential sources of autumn migration through the corridor.

## 4. Discussion

Our intensive multi-year radar study reveals that substantial bidirectional insect migration, actively directed in seasonally beneficial directions, passes through the Levantine Corridor every year. The results reflect the importance of the Levantine Corridor as an intercontinental transport pathway for the redistribution of biomass, nutrients and ecological processes. Given the geographic position of the Levant, much of this bioflow, consisting of hundreds of millions of larger insects, likely involves intercontinental movements that connect insect faunas across Europe, Asia and Africa. Furthermore, the ~70 km-wide Levantine Corridor maintains an exceptionally hospitable habitat at this latitude (compared with the deserts to the east and the Mediterranean Sea to the west). The existence of these favourable conditions implies that the chances of completing intercontinental journeys will be considerably improved for individuals traversing the Levantine Corridor. However, our surprising discovery that migration intensity is likely lower along the Levantine Corridor compared with the potential source/destination regions further north (across mainland Europe) challenges our initial idea that the corridor funnels the majority of insect migrants using the flyway linking West Asia/East Europe with Northeast Africa.

Migration will evolve and be maintained when the lifetime reproductive output accrued from moving to a new location exceeds that of remaining in the original location [1,65]. Similarly, if the balance tips and migration becomes less beneficial, then migration will be expected to disappear [66]. Thus, the seemingly lower degree of funnelling than expected, particularly during the southward autumn migration, is surprising. We posit two, not mutually exclusive, potential explanations for this apparently counterintuitive finding. First, it may be that the added costs (increased mortality risk, longer migration durations and higher energetic expenditure) of travelling long distances longitudinally (along an east–west axis) upon reaching a barrier (sea or desert) to locate the hospitable corridor outweigh the costs of latitudinal travel (along a north–south axis) across the barrier. Given the short time window that most insects have to complete their migrations [1], the increased duration of such detours might be the most important of these factors. Second, the sensory and cognitive capabilities of insect migrants may preclude the funnelling of migrants from across large longitudinal spans. In contrast to birds, which have the options of learning routes via direct experience and/or following more experienced migrants, insects using this flyway will always be engaged on a ‘maiden voyage’, with no possibility of developing route learning or following more experienced migrants [67]. These reduced navigational capabilities will likely lead to broad-front latitudinal migration across barriers and corridors alike, as is thought to be the case in painted lady butterflies [68]. Radar deployments in the Mediterranean Sea and in the desert regions to the east will be required to answer this question, although it is clearly logistically challenging to carry out such studies. However, large arrivals of insect migrants in Cyprus following migration across the sea from the Middle East [4,69], with high mortality of some of the taxa involved [12], provide some support to this contention. One or both hypotheses likely act as an evolutionary counterbalance to the potential benefits of being funnelled through the hospitable corridor. It is also quite possible that a large component of the migratory traffic along the corridor emanates from locally produced source populations, showing seasonal redistribution within the corridor rather than passage of long-range intercontinental migrants. We believe that intercontinental migrants are likely an important component, at least in some years, given that some atypical mass migrations through the corridor are known to have originated much further to the southeast (in the Arabian Desert) [4,69], but the exact patterns of contribution from long-range and local migrants remains to be resolved.

The relationship between migration intensity and meteorological conditions reveals a central role of the weather in regulating insect movements through the corridor. Some meteorological conditions, for example, strong westerly winds (blowing towards the east) in autumn or cold temperatures in spring, were found to halt migration completely. Migration rates are significantly increased on days/nights when wind directions are beneficial, i.e. blowing northward in spring and southward in autumn, clearly demonstrating that insect migrants in this region actively select favourable tailwinds to aid their long-range movements, as has been observed elsewhere [2,6,25,36]. Displacement integrates active movement (self-propelled flight speed and direction) and airflow properties (wind speed and direction) [70]. Our results clearly demonstrate meteorological selectivity as a means of migration behaviour (figure 5B,D) and strongly imply that migrants also actively propel themselves through the corridor, as clearly seen in the differences of directionality between insect movement and downwind direction (figure 4, electronic supplementary material, table S4). Data on flight headings (the direction towards which the flying insects are facing), which were not measurable using the MR1 during most of our campaign, could help elucidate the navigation process in future research. Lack of migration during the coldest temperatures in spring is characteristic of insect migration [2,7], reflecting their poikilothermic physiology and reliance on warm conditions for flight. The effects of the east–west component of the wind and humidity on migration intensity are probably interrelated. Westerly winds, blowing into Israel from the Mediterranean Sea, tend to be humid but sparse in insect migrants owing both to the sea not providing a source area for migrants and the increased chance of heavy precipitation, which can be detrimental to airborne migrants. Conversely, easterly winds, coming from the Syrian and Arabian deserts, are dry but can carry occasional large fluxes of insect migrants [4,69], directed into the corridor. These relationships drive the erratic day-to-day variation observed within season, with mass migration events interspersed with periods of very little activity in rapid succession (figure 2 and electronic supplementary material, figure S2).

Insect displacement directions during the migration seasons were significantly different from simple downwind movement on the prevailing winds, which blow towards the east/southeast. This was remarkably clear at some sites, such as the Hula Valley at night, and it clearly indicates an evolved behavioural adaptation to facilitate directional movement towards seasonally favourable habitats [14,37] and fits in with the geographic position of the Levantine Corridor between vast expanses of hostile environments. The cost of diverting away from the corridor is potentially very high (ending up in the Mediterranean Sea or the Arabian Desert), which applies strong selective force towards tightly directed movement patterns. These patterns are presumably mediated by active selection of days/nights and flight altitudes with favourable wind directions, combined with compass-mediated orientation behaviour to compensate for unfavourable drift, as observed in other large insect migrants on flyways in West Europe, East Asia, Australia and North America [2,5,14,31,37,71–73]. The fact that directionality was strongest in the Hula Valley, the lowest site in our study (60 m above sea level (a.s.l.)), and weakest in the two adjacent highest sites at Northern Golan (1000 m a.s.l.) and Eastern Upper Galilee (900 m a.s.l.) is interesting given the proximity of these sites, suggesting channelling of migrants (possibly mediated by local wind patterns) through the north–south oriented valley, which increases their ability to fly in a beneficial direction. This finding is similar to that reported in nocturnal bird migration in the Alps [74].

Based on previous data from the UK, where radar studies have demonstrated that autumn return migrations are typically considerably larger than the spring influx within the same year [33,39,71], or at least of comparable size [2], our expectation was that migrations of insect populations returning south would generally be larger than the preceding spring influx to the north. Contrary to our prediction, spring migrations were consistently larger than autumn migrations in all locations in the Levantine Corridor. This surprising result leads to many interesting questions and considerations, some of which we discuss here, but which will require substantial further investigation to answer conclusively.

Considering the MR1’s limited altitudinal detection range (up to 500 m above the ground), substantial autumn migration may have passed over undetected at higher altitudes. Considering the typically warm climate in the Levant, higher air layers are likely to be favourable for migration, potentially harbouring a large proportion of the total migration traffic. However, we suspect that vertical distribution patterns would likely be similar in the spring and autumn migration periods as examination of the profiles over the lower 500 m (figure 3C) indicates no substantial difference between spring and autumn, so this is unlikely to explain the patterns of seasonal abundance. It would be interesting to quantify the extent of migration above the radar detection threshold, nevertheless.

A second interesting possibility is that the large autumn populations of migrants heading south from higher latitudes [1,2,32,38] may experience high mortality, with many emigrants not reaching potential winter-breeding sites at lower latitudes. Until recently, there was a paucity of comparable data from lower latitudes, but recent measurements from lower latitudes in China [6,66,75], and now from Israel (the current study), indicate that numbers returning to southern parts of the annual range are smaller than those setting off from there in spring. This either suggests that southern breeding ranges are more productive (Nile delta and localized desert oases), or that mortality along the southward migration route must be substantial, designating migration as a costly and risky strategy for many insects. Losses may occur owing to windborne transport over unsuitable habitats such as the sea [12], and perhaps also owing to increased predation risk as they move into lower-latitude regions [76]. Disentangling which of these factors, acting independently or in concert, are the main drivers of the surprisingly low migration rates in the Levantine Corridor, particularly in autumn, will require further detailed ecological study of aerial insect migration through this important region.

Migratory insects are crucial pollinators for much of the wild and cultivated flora around us and they are an essential food source for hundreds of species of vertebrates [11,32,77,78]. They are also vectors for some of humanity’s most lethal diseases, some of the most destructive crop pests and the worst invasive species [16,30,79–81]. The BirdScan MR1 and similar systems are becoming increasingly common for use in biological monitoring [5,82,83], providing an exciting opportunity to substantially increase the scope of aerial insect migration research. In summary, the present study demonstrates the existence and key features of insect migration through the Middle East, with implications for ecological processes at the nexus of three continents. This characterization of the phenomenon is another step towards developing a clearer overview of insect migration-driven processes on a global scale.

## Data Availability

The datasets generated during and/or analysed during the current study are available in the Figshare repository: https://figshare.com/s/4abe912bd1adeb7352cc. Supplementary material is available online [84].
